# Simple and cost-effective way to make mobile antibiotic cement spacer: hand-made silicone mold

**DOI:** 10.1051/sicotj/2023032

**Published:** 2023-11-08

**Authors:** Quang Ton Quyen Nguyen, Ta Hoc Vo, Duc Tri Phan, Nguyen Khanh Hung Truong

**Affiliations:** 1 Department of Orthopedic Surgery, Tam Anh Hospital Ha Noi Vietnam; 2 Orthopedic and Trauma Department, Dong Nai General Hospital Dong Nai Vietnam; 3 Orthopedic and Trauma Department, Da Nang Hospital Da Nang Vietnam; 4 Orthopedic and Trauma Department, Cho Ray Hospital Ho Chi Minh City Vietnam

**Keywords:** Prosthetic joint infection, Articulating spacer, Two-stage revision, Silicone mold

## Abstract

*Background:* Two-stage exchange arthroplasty is considered the most common approach for the management of prosthetic joint infections. There has been plentiful evidence to support the superiority of the mobile spacers over the static ones. Unfortunately, articulating options are not available in our low-resource environment, which motivated us to come up with an affordable way to create a mobile cement spacer. After experimenting with a variety of materials and producing methods, we realized that silicone is a favorable material for mold building and established a simple process of making a handmade silicone mold. We demonstrate the clinical outcomes of three prosthetic joint infections by using these spacers in the hope of spreading the idea to our colleagues who work in the circumstances of a developing country. *Construction of the spacer molds*: The molds, consisting of two parts, were shaped by using high viscosity addition silicone (elite HD+ putty soft, Zhermack SpA, Italy) as material, and previously removed implants as template. They were sterilized using ethylene oxide treatment before being ready for casting antibiotic-loaded bone cement spacer. *Case report*: Three cases of prosthetic infection were treated with two-stage revision, using antibiotic-impregnated cement spacer cast in hand-made silicone molds. We sought to determine intraoperative complications, postoperative range of motion, and functional scores. All the patients were regularly followed up to identify fractures or dislocation of the spacer, and reinfection. *Results*: At the end of the follow-up, all three patients had the infection eradicated. The three patients could sit comfortably with bent knees, walk with partial weight-bearing, and achieve 75–80 degrees of knee flexion in the first week after surgery. Follow-up X-rays revealed no fractures or dislocation in any of the spacers. *Conclusion*: Silicone molds offer a simple and cost-effective alternative to costly commercial products in producing articulating spacers. Treating infected joints arthroplasty with these spacers allows for early motion and partial weight bearing and improves patient satisfaction and life quality before reimplantation without significant complications.

## Introduction

Prosthetic joint infection (PJI), representing a significant complication with an incidence of 1% to 2%, can be treated with one- or two-stage revision procedures [1]. With a success rate of over 90%, the use of antibiotic-impregnated bone-cement spacers in a two-stage technique, either articulating or non-articulating, has become the standard of care for patients with a chronic infection at the site of a total joint replacement in North America [2].

During two-stage revision with static spacer blocks, the exposure at the time of reimplantation is complicated due to secondary soft-tissue contractures. The patient will have difficulty in mobility and suffer unexpected bone loss attributable to the migration of the spacer block [3]. To overcome this problem, numerous techniques including handmade, custom-molded, and prefabricated spacers are being used to construct mobile or articulating spacers [4, 5, 6, 7]. The potential advantages of the mobile spacers over the static ones include early postoperative active movement, greater range of motion, shorter hospital stay, better functional outcome, and ease of second-stage reimplantation. Evidence supporting the superiority of mobile spacers over static ones is on the increase [8].

Due to the lack of commercial mobile cement spacers in our low resources setting and inspired by the pioneering work of our colleagues in Cho Ray Hospital [9], we developed a simple way to produce low-cost mobile cement spacers using a handmade silicone mold. We will describe here our process of experimenting with different materials and methods of fabrication, which eventually led to the choice of silicone. We will also demonstrate the promising clinical outcomes produced by our cement spacers in treating three prosthetic joint infections. And it’s the initial success that encourages us to share our ideas and experience in this effort with colleagues in need in developing countries.

## Methods

## Construction of the spacer mold

Hip implants (cemented TrendHip^®^ stem, Aesculap, Germany) and knee implants (Tricompartmental prosthesis New Wave™, Groupe Lépine, France) removed from previous prosthetic joint infections were cleaned and then sterilized in the sterrad machine before using as templates.

**Figure 1:**
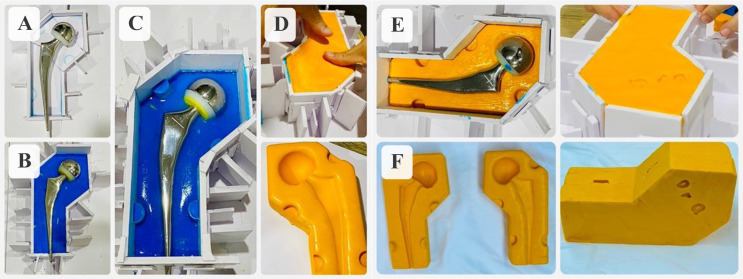
Our proposed steps to make silicone molds.

A container consisting of two equal parts, lower and upper, with dimensions matching the template size was made of a 5 mm-thick PVC foam sheet, Figure 1A. One half of the container is filled with clay and the template was secured in the clay so that its lower half would be buried under the clay surface, Figure 1B. Some gelatin was then spread over to make the surface smooth and the other half of the container was installed upon the first one, Figure 1C. The upper half was then filled up with silicone (high viscosity addition silicone, elite HD+ putty soft, Zhermack SpA, Italy), a mixture of an appropriate amount of base and catalyst in a ratio of approximately 1:1, allowing for a working time of 2 min, Figure 1D. After a setting time of 5 min 30 s, the container and clay were removed, leaving the first half of the mold with the template, which was then laid in the container again, and coated with paraffin to facilitate later removal. The container was again filled with silicone, Figure 1E. After the silicone was completely cured, the two-part mold was ready and sent for ethylene oxide sterilization, Figure 1F.

To make knee spacers, separate molds in a series of three sizes for both femoral and tibial parts were required. The molds could be cleaned and sterilized before storage for reuse.

### Making cement spacers

**Figure 2:**
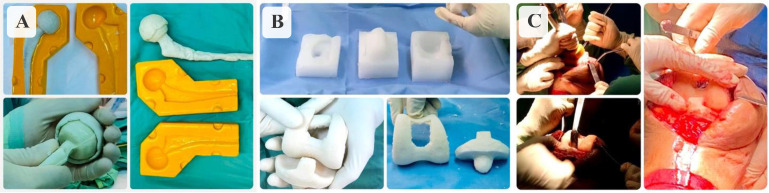
A, B. The antibiotic-loaded cement spacers are cast during surgery. C. Spacers are fixed with cement.

The silicone mold was then used for intraoperative production of antibiotic-loaded PMMA hip and knee mobile spacers. A total of two packs of cement was used to make spacers. Cement was poured into the molds during the manipulative stage (the cement does not adhere to the molds, so the use of sterile paraffin is not necessary). After several minutes, it set to form spacers with smooth surfaces, Figure 2. Another two packs of cement were used consecutively to fix the spacers to the bone.

### Surgical technique and postoperative protocol

The first step included removal of the infected prosthesis, extensive debridement, and irrigation of the joint. Tissue sampling was taken for microbiological culture and histology study. The cement spacer components with appropriate size could be implanted at the same time as implant removal with a small amount of added cement.

The antibiotic added to the cement was 3 g Vancomycin per 30 g PMMA bone cement or tailored to the antibiogram as summarized in Table 1. The stability and range of motion were tested, intra-articular drain was inserted, followed by conventional closure and sterile wound dressing.

**Table 1:** Summary of patient’s diagnosis, treatment, and outcome.

Patient	Age	Gender	Preop state	Bacteriology	Antibiotic in spacer	IV antibiotics	Oral antibiotics	AR	LEFS	Infection eradication
1	75	Male	Inf TKA	MRSA	Vancomycin 6 g	Linezolid 600 mg b.d. 6 wk	No	No	41.3%	Failed*
2	67	Female	Inf TKA	Not detected	Vancomycin 3 g + Imipenem 2 g	Imipenem 500 mg b.d. 4 wk	Cefuroxime 6 wk	Yes	60.0%	Yes
3	85	Male	Inf PHA	MRSA	Vancomycin 6 g	Vancomycin 1g b.d. 6 wk	Forlen 6 wk	No	N/A	Yes

Preop =  preoperative; IV =  intravenous; Inf = infected; TKA =  total knee arthroplasty; MRSA =  methicillin-resistant *Staphylococcus aureus*; AR = arthroplasty revision; LEFS = Lower Extremity Functional Scale; N/A: not available.

* Spacer exchange within three months, infection eradicated later.

Anterior-posterior and lateral X-rays of the joint were taken after prosthesis removal and spacer implantation to confirm the spacer position. The passive motion could be started 24 h postoperatively, ambulation was allowed with cruches. Depending on the agent, an individual protocol of intravenous antibiotics was indicated in Table 1, for at least six weeks until infection control was confirmed with clinical observation and progressive decline of erythrocyte sedimentation rate and C-reactive protein. Discharged patients were administered a subsequent oral antibiotic coverage for six weeks.

All three patients were regularly followed up for clinical assessment including range of motion, functional scores using the lower extremity functional scale (LEFS), radiographic and laboratory examinations.

## Results

**Figure 3:**
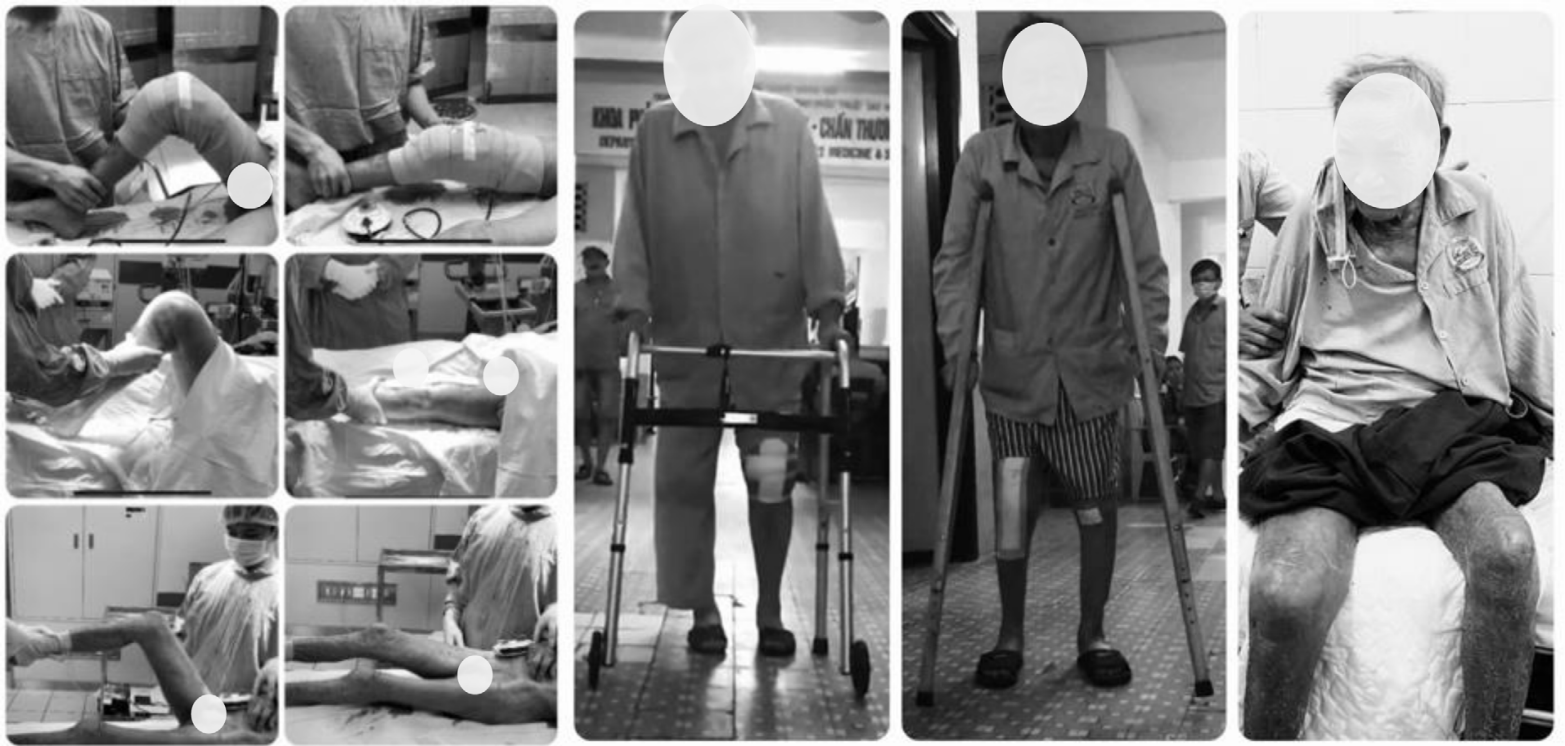
A. Intra-operative range of motion, B. Patients walking/sitting in first week after surgery.

Three patients with a diagnosed prosthetic joint infection, one hip and two knees, were treated for a period of 18 months using mobile antibiotic-loaded spacers. During the first 6 weeks of surgery, the patient with hip spacer was allowed for only a non-weight bearing to ensure sufficient bone healing, while he could sit comfortably with bent knees, and subsequently a partial weight bearing with a walking frame. The other two patients with knee spacers were able to walk with partial weight-bearing and achieved 75–80 degrees knee flexion at week one after surgery, Figure 3.

**Figure 4:**
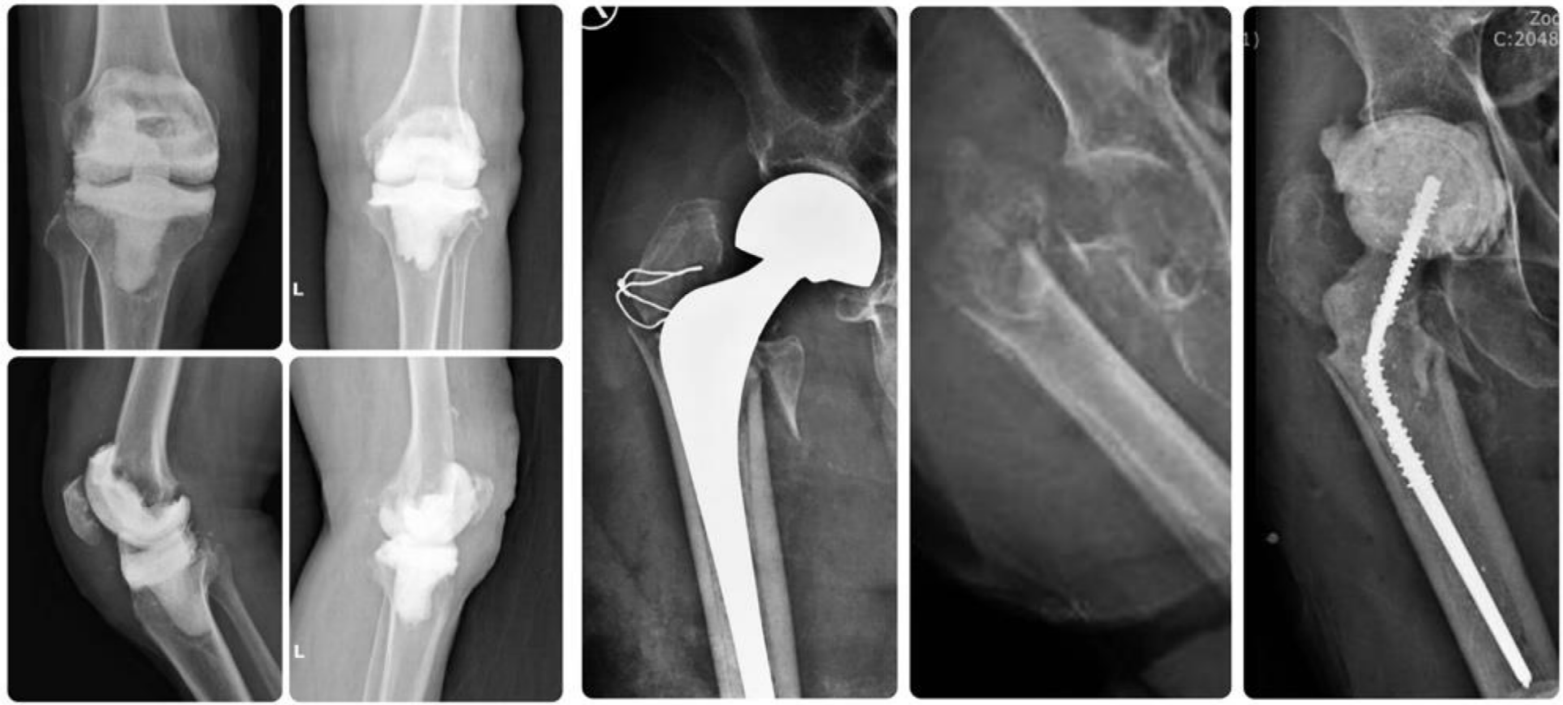
Postoperative X-rays.

Follow-up X-rays revealed no fractures or dislocation of the spacer in any case, Figure 4. In one patient, a spacer exchange was necessary within three months after the primary spacer implantation due to recurrent infection.

**Figure 5:**
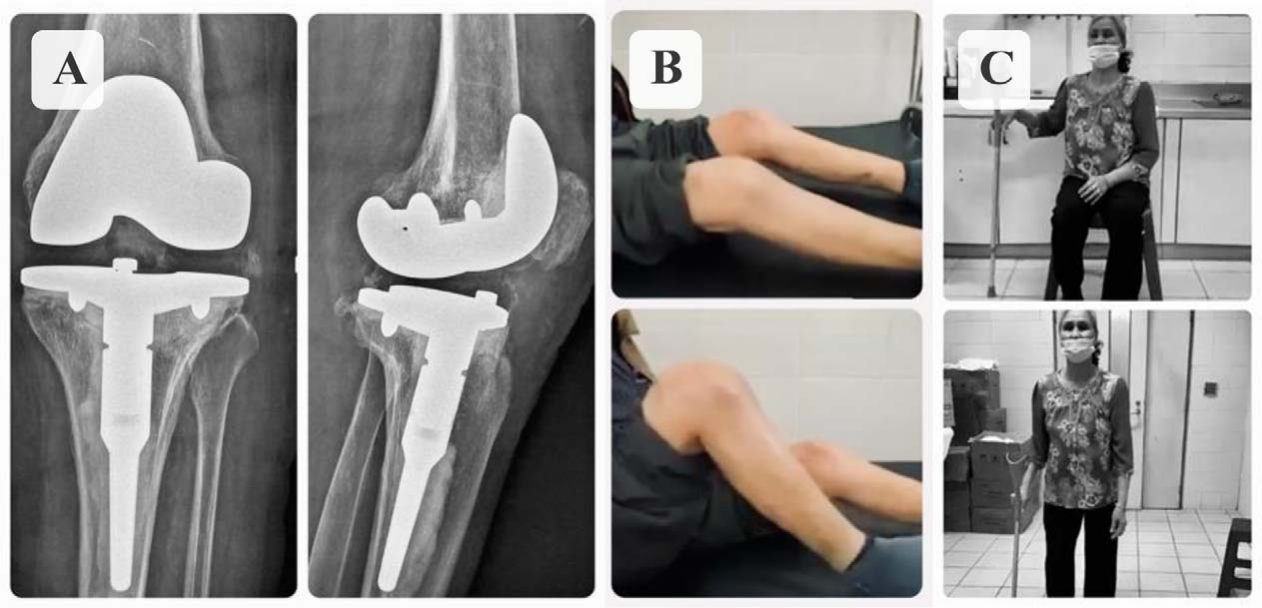
A. Postoperative X-ray of revision surgery by hinge prosthesis, B. Patient with cement spacer in place and C. Patient undergoes revision at final follow-up.

One patient with a knee spacer decided to have second-stage total knee revision 3 months after the first-stage surgery, but the other refused to undergo revision arthroplasty for fear of having another operation, which resulted from his bad experience with the previous prosthetic joint infection. At the final follow-up at month 12, the patient with revision arthroplasty had no pain and the patient with knee spacer had mild pain, they were both comfortably walking with assistive devices, Figure 5. LEFS of the two patients are 60.0%, and 41.3% respectively, and both had their infection eradicated. The patient with a hip spacer was not appropriate for revision surgery due to his age and general health status.

## Discussions

The two-stage protocol for the revision of infected total knee prosthesis with the insertion of antibiotic-impregnated cement spacers was first described by Insall [10]. Since the success rates of this strategy exceeded 90%, it has been widely adopted [11].

Spacers can be static or mobile, which have demonstrated similar ability to eradicate infections in the treatment of prosthetic joint infections. Today, the available literature recommends that surgeons take into account the potential benefits of mobile spacers such as early active movement, greater range of motion, shorter hospital stay, better functional outcome, lower unexpected bone loss, and more favorable second-stage reimplantation [1, 3, 8].

Several techniques to construct mobile spacers and their effectiveness have been reported: handmade, custom-molded, prefabricated, or 3D printing-assisted spacers [12, 13, 14, 7]. Prefabricated or 3D printing-assisted techniques help create a well-shaped spacer thereby permitting mobility, but high costs prevent them from being available in countries with low medical resources. Besides, the incorporated antibiotics cannot be tailored to the individual patient and the dose is often suboptimal for the treatment of chronic infection.

Traditional approaches like intraoperative handmade spacers are quite commonly used [12, 15, 16]. Nevertheless, making spacers in this way is time-consuming and the final products are often inconsistent in shape, surface and congruency, which may lead to limited motion, excessive wear, and risk of instability.

Su et al. have proposed a handmade articulating spacer by impression-taking technique, which is cost-effective and versatile [6]. However, this method results in thin molds, which can possibly deform during the spacer casting process and would probably not be suitable for creating hip spacer mold. Another potential drawback of this technique is that it does not allow the cement to expand during polymerization inside a closed mold to create a smooth surface on the final cement spacer.

Aluminum molds for making cement spacers have also been developed by other authors. Although it is considered simple, cheap, and easy to apply, the two methods of producing aluminum molds – computerized CNC machine (DATRON Booth N-6021) [9] and computerized numerical-control sinking machine (DMU 70 eV-process) [17] – are only accessible in well-industrialized regions, and not affordable to process in small quantities. In fact, we have contacted many local manufacturers but the answer was always the same they do not have a CNC machine for processing metal with a thickness of more than 9 mm.

**Figure 6:**
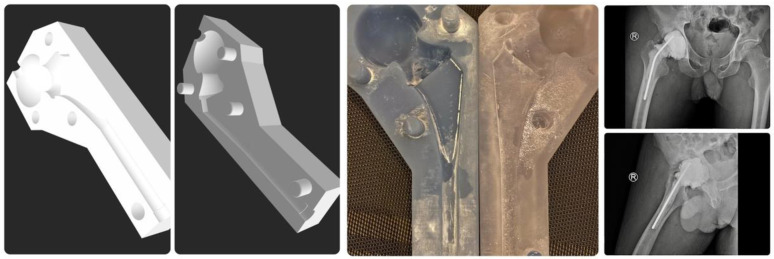
3D design, 3D printing resin mold, and cement spacer.

In search of a more viable method, we have attempted 3D design and 3D printer and were able to make very smooth surface resin molds with the digital light processing technology, Figure 6. The low elasticity and high embrittleness of this material combined with the expansion of PMMA cement during polymerization makes it difficult to remove the spacer from the mold, resulting in the breakage of the resin mold and making this method quite costly for producing spacers.

After doing some trial and error studies, we decided to use a very popular material: silicone in a simple method of shaping two-part molds. This technique can effectively cast any complex shapes with an incredible resolution. Silicone with high elastic recovery allows for removing the cement spacer easily out of the mold even without any lubricant.

The first prototype, made of industrial silicone, drew criticism from our colleagues of the safety and the risk of toxicity to patients. The latter were made of additional silicone, an impression material in dentistry, which is described as having very low shrinkage on setting, low flow, and high elastic recovery, and already licensed for medical use [18]. Another advantage of silicone A is that it is available almost anywhere there is a dental facility. In addition, this material is inexpensive, costing only 70 dollars to make a mold.

We used cemented TrendHip^®^ stem and tri-compartmental prosthesis New Wave™ as templates due to their popularity and availability in our institute. However, this methodof silicone mold-making can be applied with any other implant.

To the best of our knowledge, the only commercially available silicone molding system is the StageOne knee cement spacer molds (Biomet Orthopaedics Inc., Warsaw, IN). These molds are designed for single use, which makes it quite costly, and again this is an inaccessible option in developing countries.

A variety of disinfectants – including neutral glutaraldehyde, acidified glutaraldehyde, neutral phenolated glutaraldehyde, phenol, iodophor, and chlorine dioxide may be used to disinfect additional silicone [18]. In a study to evaluate the effect of three common sterilization techniques on the mechanical properties of silicone rubbers, Emilie Gautriaud et al. [19] concluded that ethylene oxide sterilization did not have a significant negative effect on the properties of commercial silicone rubbers. For this reason, our silicone molds were treated with ethylene oxide.

In this study, we report only the preliminary results of using handmade silicone molds for casting cement spacers for the management of three cases of prosthetic joint infection with a short follow-up time. There was one recurrence of infection, whose cause could not be identified. In the second surgery, we did a more aggressive debridement, replaced the old spacer with a new one, and administered oral antibiotics for 6 weeks. As a result, the infection was successfully eradicated.

In summary, the ability to walk with partial weight-bearing, and early range of motion without fractures or dislocation are encouraging results. Our mold-making method does not require hi-tech equipment, allows flexibility in using antibiotics, and can be applied to different joints. Silicone molds can be cleaned, resterilized, and reused, helping produce spacers with smooth surfaces, good stability, and low cost. It is highly feasible for any hospital to apply.

## Limitations of the study

The present study has exposed the following limitations: the small number of patients, absence of a control group with other spacers, no data on the biomechanical characterization of the spacer, short follow-up time, and final clinical outcomes which remain to be determined because only one patient come back for arthroplasty revision. Prospective randomized controlled trials with a larger number of cases and longer follow-up periods should be carried out to confirm the effectiveness of articular spacers made with handmade silicone molds.

## Conclusion

Silicone molds offer a simple and cost-effective alternative for making articulating spacers to serve the treatment of patients where commercial products are unavailable or unaffordable. Arthroplasty for treating infected joints with these spacers promises to make a substantial contribution to the effective eradication of infection, allowing for early motion and partial weight bearing, patient satisfaction, and life quality prior to a more favorable second-stage reimplantation of a prosthesis.

## Conflicts of interest

The authors declare that they have no conflicts of interest inrelation to this article.

## Funding

This research did not receive any specific funding.

## Ethical approval

Ethical approval was not required.

## Informed consent

Ethical approval was not required.

## Authors contributions

Nguyen Quang Ton Quyen put forward the ideas and performed all the operations, wrote the first draft of the report; Vo Ta Hoc, Phan Duc Tri: performed clinical examination, dealt with X-ray images and patient follow-up, assisted in editing and completing the report, Truong Nguyen Khanh Hung: provided key comments to the report and final editing. All the authors have read the manuscript and agreed with its final report.
